# Metabolic Modeling to Interrogate Microbial Disease: A Tale for Experimentalists

**DOI:** 10.3389/fmolb.2021.634479

**Published:** 2021-02-18

**Authors:** Fabrice Jean-Pierre, Michael A. Henson, George A. O’Toole

**Affiliations:** ^1^ Department of Microbiology and Immunology, Geisel School of Medicine at Dartmouth, Hanover, NH, United States; ^2^ Department of Chemical Engineering and Institute for Applied Life Sciences, University of Massachusetts, Amherst, MA, United States

**Keywords:** metabolic modeling, metabolite cross-feeding, gut microbiome, cystic fibrosis, drug discovery

## Abstract

The explosion of microbiome analyses has helped identify individual microorganisms and microbial communities driving human health and disease, but how these communities function is still an open question. For example, the role for the incredibly complex metabolic interactions among microbial species cannot easily be resolved by current experimental approaches such as 16S rRNA gene sequencing, metagenomics and/or metabolomics. Resolving such metabolic interactions is particularly challenging in the context of polymicrobial communities where metabolite exchange has been reported to impact key bacterial traits such as virulence and antibiotic treatment efficacy. As novel approaches are needed to pinpoint microbial determinants responsible for impacting community function in the context of human health and to facilitate the development of novel anti-infective and antimicrobial drugs, here we review, from the viewpoint of experimentalists, the latest advances in metabolic modeling, a computational method capable of predicting metabolic capabilities and interactions from individual microorganisms to complex ecological systems. We use selected examples from the literature to illustrate how metabolic modeling has been utilized, in combination with experiments, to better understand microbial community function. Finally, we propose how such combined, cross-disciplinary efforts can be utilized to drive laboratory work and drug discovery moving forward.

## Introduction

Bacteria possess an incredible arsenal of tools allowing them to thrive in diverse environments. Such an adaptive capacity partly stems from their ability to metabolize nutrients that are present in their surroundings thus allowing them to sustain microbial growth in complex environments (Görke and Stülke, 2008). Multiple reports have revealed how metabolic features are important for bacterial persistence, virulence and drug tolerance during the infection process (Eisenreich et al., 2015; Peng et al., 2015; Eisenreich et al., 2017; Adamowicz et al., 2018; Herrero-Fresno and Olsen, 2018; Sprenger et al., 2018; Crabbé et al., 2019; Perinbam et al., 2020). However, obtaining a complete portrait of how metabolism and metabolic interactions can impact these diverse bacterial phenotypes is still a matter of active research (Zuniga et al., 2017).

Observations dating from as early as the 17th century examining dental plaque support the idea that microorganisms often live as polymicrobial, biofilm-like communities and not as single-species in a planktonic environment (Leewenhoeck, 1684; O’Toole, 2016). Such complex biofilm communities are composed of microbial species that have the potential to interact through exchange of metabolites, ultimately shaping the microbial ecosystem (Zuniga et al., 2017; Antoniewicz, 2020).

Culture-based methods have helped us understand phenotypes associated with specific microorganisms. In contrast, the presence of complex microbial community diversity and spatiotemporal organization existing in some environments, such as in the oral cavity or the human gut, makes it inherently difficult to obtain a clear picture of how metabolic interactions among community members influence, and are being influenced by, their local environment (Peters et al., 2012; Miller et al., 2019). The advent of culture-independent techniques have paved the way to a new golden age in the understanding of microbial biology and ecology, revealing the complexity existing within complex microbial communities and their impact on human health, agriculture and the environment (Kinross et al., 2011; Chaparro et al., 2012). Indeed, it has been shown that diseases such as colorectal cancer and inflammatory bowel disease can be associated with imbalances in the microbiome, also known as dysbiosis (Gilbert et al., 2016). While the use of next-generation sequencing technologies now make it clear that many human niches are colonized by multiple microbial species and taxonomic/functional features can be associated with healthy and disease states, knowing only “who is there” and keeping a “gene catalogue” is not sufficient to probe functional metabolic roles of each member present in a community (Frioux et al., 2020). Furthermore, it is likely that there are instances when communities, classified as “different” based on sequencing data share common metabolic features. For instance, a study published by Jorth and colleagues looking at microbial communities associated to periodontal disease reported that no clear polymicrobial community structure could be associated with disease due to high interpatient variability (Jorth et al., 2014). However, assessing the metabolic capacities of these communities revealed that disease-associated microbial clusters maintained a conserved functional profile (Jorth et al., 2014).

Although metagenomics can alleviate some of the shortcomings of 16S-based rRNA gene amplicon studies by providing an overview of metabolic potential and metabolomics can identify the abundant, accumulated metabolites in a community, these approaches alone are still not sufficient to probe essential metabolic functions and metabolite-based interactions among microbes in a community (Quince et al., 2017; Frioux et al., 2020). The importance of developing novel strategies to understand how metabolic interactions drive community structure and function is further highlighted by recent reports indicating that such microbial or host-driven metabolic interactions can modulate drug resistance and tolerance (Peters et al., 2012; Crabbé et al., 2019; Crabbé et al., 2019; Orazi and O’Toole, 2019).

Therefore, we propose that *metabolic modeling* is one such tool that exploits existing amplicon, metagenomics and metabolomics data to generate hypotheses that can complement and help drive experimental studies, thereby validating the computational interrogations of these existing datasets. Metabolic modeling is an *in silico* predictive mathematical modeling approach that leverages genome-scale metabolic model (GEM) reconstructions of cellular metabolism derived from genomic annotations (Biggs et al., 2015; Zomorrodi and Segre, 2016; Zampieri et al., 2019). Since the *in silico* reconstruction of the first GEM of *Haemophilus influenzae* in 1999 (Edwards and Palsson, 1999) hundreds of novel GEMs have been generated (through automatic, semi-automatic or manual means) as new genomes are being sequenced (Gu et al., 2019). This approach has been used to drive biochemical knowledge of biological systems by 1) translating functional annotation information into metabolic predictions, 2) probing metabolic features involved in metabolite production, and 3) advancing the understanding of metabolic interactions among microbial species with their host (Thiele et al., 2013; Simeonidis and Price, 2015; Kumar et al., 2019). As a growing number of high-quality GEMs are available through several databases (Gu et al., 2019; Noronha et al., 2019; Seaver et al., 2020) and can be used to generate predictions of community biological functions through various modeling tools such as SteadyCOM, CASINO, COMET and BacArena (Harcombe et al., 2014; Shoaie et al., 2015; Bauer et al., 2017; Chan et al., 2017; Altamirano et al., 2020), metabolic modeling represents a powerful approach to interrogate complex community metabolic interactions involved in health and disease (Gu et al., 2019; Kumar et al., 2019). Furthermore, metabolic modeling methods have the capacity to guide laboratory work by integrating and making predictions based on 16S rRNA gene, metagenomics, and metabolomics datasets (Rai and Saito, 2016).

With recent evidence pointing towards the impact of polymicrobial metabolic interactions shaping human health, we review here some of the latest research leveraging metabolic modeling and genome-scale metabolic reconstructions used to interrogate metabolic interactions among the members of complex microbial communities in health and disease. We first start by surveying studies in the context of the human gut microbiome for which metabolic modeling has been mainly applied, and then transition to the case of cystic fibrosis (CF), a genetic disease where persons with CF (pwCF) accumulate thick secretions (mucus) in their airways, which creates an ideal nutrient-rich environment wherein pathogens can thrive, ultimately leading to high morbidity and mortality (Boucher, 2007; O'Sullivan and Freedman, 2009; Surette, 2014). We also present findings indicating how metabolic modeling could be used to develop new antimicrobial drugs. We review this literature through the lens of experimentalists who can integrate such modeling data to focus their efforts on understanding the role of metabolic interactions in driving microbial community structure and function.

### Metabolic Modeling to Probe Microbial Interactions and Understand Disease

**FIGURE 1:**
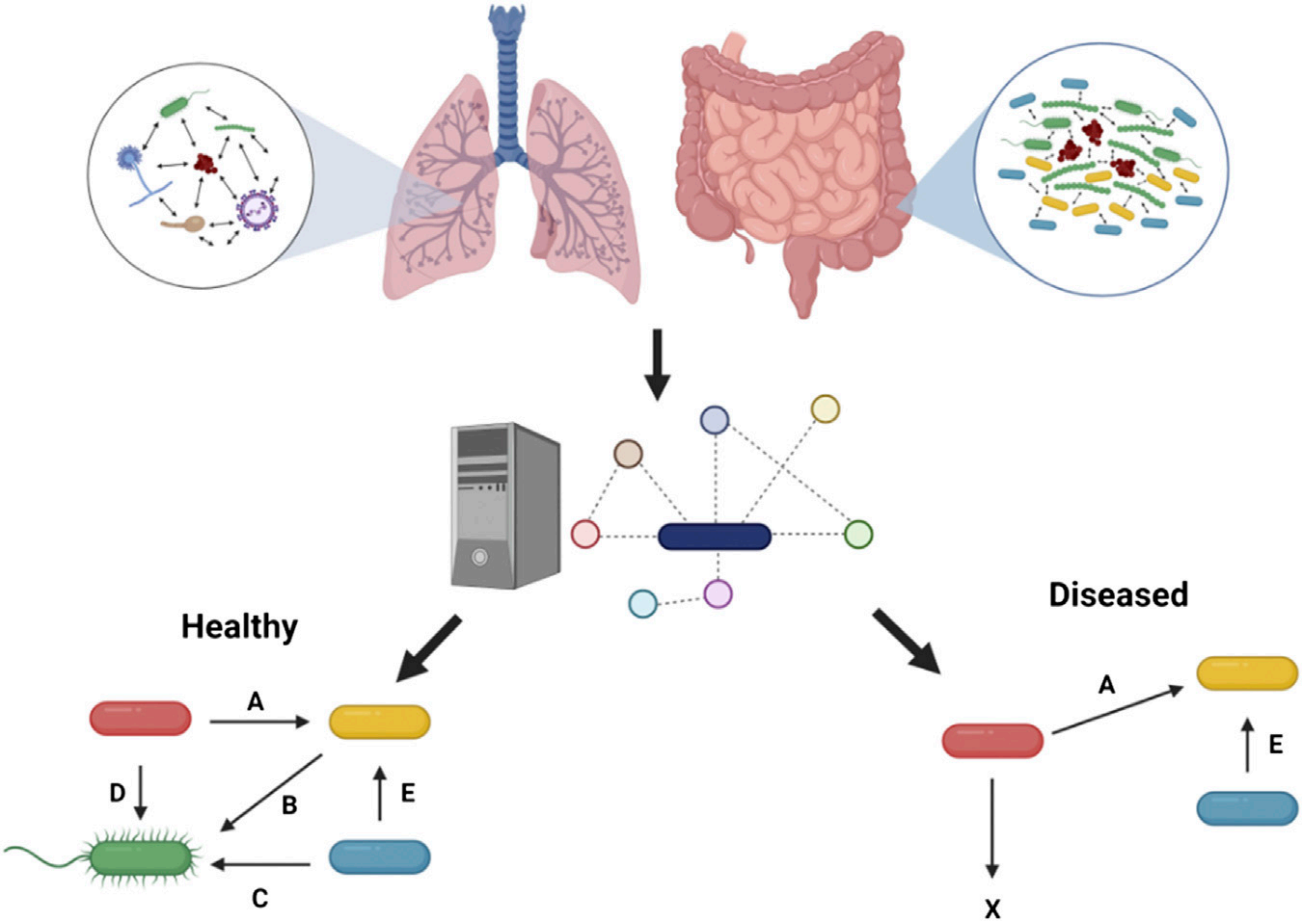
Metabolic modeling to understand health and disease. Microbial interactions observed in the human gut and in the context of chronic lung disease such as in pwCF can be predicted through metabolic modeling to pinpoint metabolic cross-feeding interactions (denoted by letters) driving community structure and associated with healthy or diseased states. These predictions are facilitated by the capacity of this *in silico* approach to integrate *in vivo*-like nutritional and physico-chemical parameters, and ultimately help guide experimentation. Figure designed using BioRender

Human health can be profoundly impacted by the presence of microbial communities, which must include taking into account how the individual microbes in these communities interact with each other (Peters et al., 2012; Miller et al., 2019; Orazi and O’Toole, 2019). Although some of these metabolic interactions among microbes in a community can be beneficial to maintain health, for example the presence of microbial communities capable of digesting complex molecules in the gut (Backhed et al., 2005), some chronic infectious diseases, such as periodontitis, CF and diabetic foot ulcers, have been shown to be driven by polymicrobial communities (Dowd et al., 2008; Lebeaux et al., 2014; O’Toole, 2018; Miller et al., 2019). We start here with a discussion of findings using metabolic modeling to study microbial community function, largely with a focus on communities relevant to disease, especially in the case of the human gut (Figure 1). Many of these studies validate the modeling findings by using *in vitro* experimental models.

As mentioned above, one of the research areas benefiting from metabolic modeling is the study of the human gut microbiome (Shoaie and Nielsen, 2014; Ji and Nielsen, 2015; Kumar et al., 2019; Sen and Oresic, 2019). The human gut is composed of trillions of microbial cells of diverse species that have an important impact on human health as they can perform a number tasks, including but not limited to, metabolizing complex molecules that cannot be degraded by the eukaryotic cells in the intestine to provide energy to colonic cells and playing a role in immune function (Bull and Plummer, 2014; Shoaie and Nielsen, 2014). Short chain fatty acids (SCFAs) produced by the gut microbiota are absorbed by the intestinal epithelial cells and impact several aspect of human health, from energy regulation to the immune programming (Comalada et al., 2006). The concentration of the SCFAs acetate, butyrate and propionate in the human body can vary depending on the diet (den Besten et al., 2013). While acetate is the SCFA mainly found in the blood and contributes to several metabolic functions such as lipogenesis and glyconeogenesis (Comalada et al., 2006), butyrate is preferred by human colonocytes for high energy production (Hamer et al., 2008; Dumas, 2011). Imbalances in the metabolism of SCFAs characterized by low butyrate production have been associated with human diseases such as inflammatory bowel disease, type 2 diabetes, obesity and other pathologies (Kumar et al., 2019).

SCFAs have also been found to be important players in the maintenance of a healthy gut microbiome through microbial interactions (Shoaie and Nielsen, 2014). For example, the presence of enzymatic degradation activity of dietary fibers into SCFAs by microbial communities in the human gut is critical to maintain health (Koh et al., 2016). To better understand how microbial interactions impact human and microbial metabolism, key human gut bacteria, that is, *Bacteroides thetaiotamicron, Eubacterium rectale* and *Methanobrevibacter smithii* (part of the Bacteroides, Firmicute and Euryarchaeota phyla, respectively) previously identified to be SCFA producers (Holmes et al., 2012) have been studied through metabolic modeling. Shoaie and colleagues reconstructed GEMs of these three bacteria by coalescing multiple biochemical reaction databases with manual curation of metabolic pathways (Shoaie and Nielsen, 2014). This work resulted in the creation of three GEMs that were used to 1) predict the metabolic landscape of metabolites that are produced and consumed within that community and 2) infer microbial biomass abundance based on known metabolites present in the environment. This analysis predicted microbe-microbe and host-microbe interactions via metabolites using a compartmentalization metabolic framework, that is, all the predicted metabolic reactions occurring within an organism were modeled and the produced metabolites allowed to be exchanged freely through a shared compartment (Biggs et al., 2015). The authors observed that acetate was the main metabolite predicted to be cross-fed between *E. rectale* and *B. thetaiotamicron* when modeled *in silico* in the germ-free gut extracellular space. Also, the *B. thetaiotamicron*-derived acetate was predicted to be driving the production of butyrate by *E. rectale*; butyrate was in turn consumed by the gut epithelial cells. These predictions are in agreement with published experimental data whereby germ-free mice co-infected with both species resulted in changing concentrations of these metabolites (Mahowald et al., 2009; Sen and Oresic, 2019). The inclusion of transcriptomic data from infected mice in the model also allowed the investigation of *E. rectale* and *B. thetaiotamicron* gene expression changes in the presence of each other (*vs*. monospecies), and could serve to validate the modeling data. *In silico* modeling indicated that *E. rectale* increases the utilization of the amino acid glutamine, whereas pathways for polysaccharide utilization in *B. thetaiotamicron* were hypothesized to increase when *E. rectale* is present; predictions that are agreement with previously published transcriptomic data (Mahowald et al., 2009). Therefore, in this instance, metabolic modeling represents a useful approach to understand how microbial interactions can impact SCFAs production and ultimately gut health.

The growing number of studies using germ-free mice models in combination with metabolomics and next generation sequencing constitute rich data sets that can feed metabolic predictions and ultimately better probe metabolic features impacting human health (Ji and Nielsen, 2015; Franzosa et al., 2019; Lavelle and Sokol, 2020). Thus, understanding how diet can impact gut health and microbial community structure through modeling approaches constitute a powerful approach that can drive experimentalist to build models to validate testable hypotheses and perhaps better understand chronic gut disease. As more GEMs are constructed and becoming available (Magnúsdóttir et al., 2017) a growing number of studies are building more complex community metabolic models to understand the interactions occurring among microbes and the host in the gut (Kumar et al., 2019).

In one of the most extensive studies modeling multiple microbial interactions in the human intestine, Henson & Phalak leveraged semi-curated genome-scale metabolic reconstructions (described above as GEMs) of 28 bacterial species detected in the human intestine (Henson and Phalak, 2018) available through the Virtual Metabolic Human database (Noronha et al., 2019). This model included a total of 22,203 genes, 26,867 metabolites and 35,031 reactions and revealed that optimal metabolic community growth rate resulted in decreased gut microbial diversity and the enrichment of just a few bacterial genera such as *Escherichia, Enterobacter* and *Citrobacter* (part of the Enterobacteriaceae family), all known to be detected in high numbers in the gut from clinical studies of patients with inflammatory bowel disease (IBD) (Rhodes, 2007; Saleh and Elson, 2011; Kaakoush et al., 2012). Furthermore, optimal community growth rate predictions resulted in different SCFA production levels (acetate overproduction and no butyrate production) compared to published experimental data (den Besten et al., 2013) suggesting that *in vivo* optimal community growth objectives might be kept in check to ensure appropriate SCFA production and healthy gut homeostasis. For example, the authors observed that reduced community growth rate, (i.e. not at its maximal value) increased microbial diversity and produced SCFA synthesis rates more consistent with those observed *in vivo* (den Besten et al., 2013). Interestingly, a recent report examining the changes in microbial community composition in healthy and IBD individuals by 16S rRNA gene amplicon sequencing observed a decrease in microbial diversity and an increase in Enterobacteriaceae abundance in the gut of diseased *vs*. healthy individuals (Alam et al., 2020). This approach of using sub-optimal growth rates in the context of communities is a fundamentally different approach compared to traditional, single-species metabolic modeling approaches that assume maximal growth rates (Biggs et al., 2015); thus, in this case experimental data helped to fine-tune the modeling approach. Moreover, metabolic modeling represents a complementary tool to experimental observations to better understand the metabolic perturbations that might be associated with imbalances in microbial diversity and abundance.

While metabolic modeling can be used to probe interactions hypothesized to drive gut health and microbial community structure, this approach can also be used to understand gut microbial imbalances in the context of infection. The presence of a healthy gut microbiome has been reported to be important for protection against pathogenic species such as the Proteobacterium *Clostridioides difficile* (Lagier, 2016; Leslie et al., 2019). Furthermore, other risk factors such as antimicrobial treatment have been implicated in the emergence of *C. difficile* infections (CDIs) (Lagier, 2016). As CDIs have been reported to cause between 15,000 and 30,000 deaths annually in the US (Lessa et al., 2015) and many healthy individuals are asymptomatic carriers of *C. difficile* (Furuya-Kanamori et al., 2015), it is important to understand how gut metabolic interactions can impact colonization resistance against *C. difficile*. By modeling microbial interactions in multispecies biofilms through the use of GEMs of *B. thetaiotaomicron* (Bacteroidetes), *Faecalibacterium prausnitzii* (Firmicute), *Escherichia coli* (Proteobacteria) and *C. difficile*, Phalak and Henson observed that among the metabolites that were predicted to be produced and cross-fed, that is, acetate, ethanol, formate and succinate, only formate produced by both *F. prausnitzii* and *E. coli* as well as *B. thetaiotaomicron*-derived succinate were hypothesized to be positively driving *C. difficile* abundance. Moreover, *in silico* removal of both organic acids would block this pathogenic microbe’s expansion (Phalak and Henson, 2019). Interestingly using a co-colonization gnobiotic mouse model, Ferreyra and colleagues observed that *B. thetaiotaomicron*-derived succinate can be utilized by *C. difficile* to allow this pathogen to thrive in the gut (Ferreyra et al., 2014), a finding consistent with the metabolic modeling data. Phalak & Henson also observed that acetate produced by *C. difficile* was hypothesized to be the only metabolite cross-fed to *F. prausnitzii* in a mixed community. However, the hypothesized cross-feeding of metabolites remain to be experimentally validated, perhaps through the development of a four-species community or using a simplified community in a mouse model.

Metabolic modeling has also been used to understand other human diseases such as CF where the lung environment constitutes an ideal locale for disease-causing pathogens to colonize, resulting in chronic infections and promoting negative clinical outcomes (Lipuma, 2010; Surette, 2014). The chronic nature of these infections is due to the presence of polymicrobial biofilm-like communities that exhibit both resistance and tolerance (collectively called recalcitrance) to front-line drugs targeted at these infections (O'Toole, 2018; Orazi and O'Toole, 2019; Vandeplassche et al., 2019). While the development of novel antimicrobials has resulted in the increase in life expectancy for pwCF, these individuals are still burdened with significant clinical symptoms (Limoli et al., 2016; O’Toole, 2018; Bevivino et al., 2019; Limoli and Hoffman, 2019). Several groups have attempted to identify the etiology of chronic CF lung disease, but this remains a topic of active research (O'Toole, 2018). Multiple groups have experimentally reported metabolic interactions among bacterial species that are prevalent and abundant in the CF airway (Crabbé et al., 2014; Orazi and O'Toole, 2017; Tavernier et al., 2017; Tavernier et al., 2018; Crabbé et al., 2019; Orazi and O’Toole, 2019; Orazi et al., 2019; Vandeplassche et al., 2019; Camus et al., 2020; Orazi et al., 2020). Based on these observations, Henson *et al.* tested the hypothesis that it would be possible to infer microbial abundance and map key metabolic interactions that potentially drive CF lung disease by modeling 17 of the most abundant species that encompass most of the 16S rRNA gene reads from three published microbiome studies (Henson et al., 2019). Interestingly, they reported that metabolic simulations performed using different *in silico* lung environments could reproduce experimentally observed 16S rRNA gene normalized reads detected in the CF airway including the infrequent microbial species *Achromobacter, Escherichia* and *Burkholderia*. Metabolic modeling approaches also allowed for the observation of interpatient community composition heterogeneity (β-diversity) that is common in pwCF (Filkins et al., 2012; Price et al., 2013; Carmody et al., 2018). The model accurately predicted that *Prevotella*, *Pseudomonas* and *Streptococcus* would dominate the polymicrobial community, an observation that is strong agreement with multiple experimental studies highlighting the impact of these pathogens on the CF airway (Lipuma, 2010; Scott and O’Toole, 2019; Thornton and Surette, 2020). Furthermore, Henson *et al.*, reported that predicted metabolic interactions in some communities were mainly driven by *Pseudomonas* and/or *Streptococcus*, which are highly abundant microbes capable of metabolizing nutrients such as organic acids, amino acids and secreted alcohols. For instance, the presence of rare pathogens was hypothesized to be driven by 1) acetate, formate and L-lactate produced by *Streptococcus* and consumed by *Escherichia*, 2) acetate, alanine and formate cross-fed from *Pseudomonas* and *Streptococcus* to *Burkholderia*, and to a lesser extent 3) alanine, threonine and L-lactate metabolite consumption by *Achromobacter*.

While some studies have focused on the metabolic interactions of microbes found in the CF airway, most of them have included two microbial species due to complex experimental methods required to culture multiple microbes in a mixed community *in vitro* (Filkins et al., 2015; Flynn et al., 2016; Scott et al., 2019; Camus et al., 2020; Li et al., 2020). However, metabolic modeling represents a useful tool to interrogate metabolic dependencies of more complex multispecies communities detected in pwCF. Furthermore, recent reports indicate that defects in the cystic fibrosis transmembrane regulator impacts mitochondrial metabolism and results in an imbalance of metabolites produced by immune cells, with possible impacts on microbial species such as *P. aeruginosa* and *S. aureus* (Gabryszewski et al., 2019; Riquelme et al., 2019; Riquelme et al., 2020a; Riquelme et al., 2020b).

The findings presented here further support the idea that metabolic modeling represents an effective approach to better understand how metabolic interactions among microorganisms can impact human health and disease such as in the gut and the CF airway. That is, by integrating experimental data from these studies, metabolic modeling can generate predictions about how polymicrobial communities might interact and a serve as a tool to develop hypotheses to be tested in the laboratory and unlock insight as to which metabolites might drive changes from healthy to diseased states.

### Metabolic Modeling as a Tool to Identify Novel Antimicrobial Targets

**FIGURE 2:**
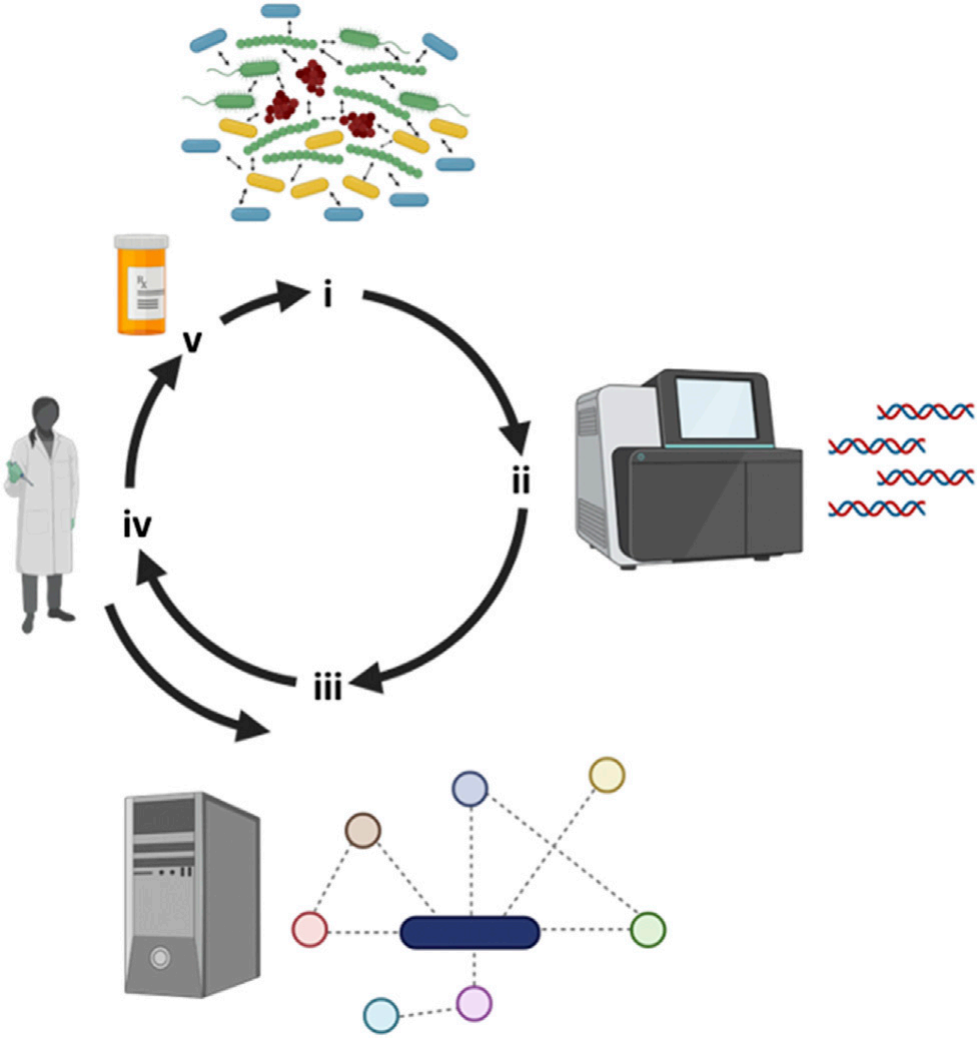
Iterative workflow between clinical observations, metabolic modeling and experimental work. (**i**) Several recalcitrant biofilm-based chronic infectious diseases are polymicrobial in nature and next-generation tools have allowed us to (**ii**) identify “who is there”. (**iii**) Metabolic modeling can leverage sequencing information by reconstructing metabolic networks and predicting key metabolites responsible of driving community structure and function. (**iv**) Metabolic predictions can then be experimentally tested and the model fine-tuned with the integration of this new experimental data. (**v**) Through modeling predictions, novel drugs can then be designed or repurposed to negatively impact polymicrobial communities. Figure designed using BioRender.

Targeting pathways implicated in the metabolism of essential nutrients utilized during infection might represent a novel approach for the identification and or development of antimicrobials that can interfere with microbial growth in the context of human disease (Figure 2) (Flynn et al., 2016; Flynn et al., 2020). The studies below use a combination of metabolic modeling and experimentation with a focus on individual microbes to better understand responses to antimicrobial therapy. This *in silico* method has also proven to be useful to understand how metabolic reprogramming can impact gene essentiality in microorganisms exposed to various antimicrobials, and thus may also help in the identification of novel drug targets.

Several groups have successfully applied metabolic modeling approaches to understand the global metabolic changes associated with microbial drug exposure, including for *P. aeruginosa* and polymixin (Zhu et al., 2018) and *Acinetobacter baumanii* exposed to colistin (Presta et al., 2017). While remaining to be experimentally validated, these *in silico* predictions revealed that exposure to antimicrobials resulted in known non-essential genes becoming essential in the presence of the drug, a phenomenon sometimes referred to as conditional or synthetic lethality. Although experimental approaches can be used to identify genes and/or synthetic lethal relationships that could represent novel drug targets (Côté et al., 2016), metabolic modeling has the advantage of facilitating the identification of such pathways and/or metabolic interactions by computationally inactivating the pathways, thus potentially helping to guide laboratory work to facilitate antimicrobial development (Raman et al., 2018).

Cesur and colleagues investigated predicted drugs targeted against *Klebsiella pneumoniae*, part of the ***E*** *nterococcus faecium*, ***S*** *taphylococcus aureus*, ***K*** *lebsiella pneumoniae*, ***A*** *cinetobacter baumannii*, ***P*** *seudomonas aeruginosa*, and ***E*** *nterobacter* species (ESKAPE) multidrug resistant group, for which cephalosporin and carbapenem resistance have been identified in part due to widespread acquisition β-lactamase-coding and carbapenemase-coding genes (Cesur et al., 2020; De Oliveira et al., 2020). *Klebsiella* species are capable of causing pneumonia and are associated with increased morbidity and mortality (Bengoechea and Sa Pessoa, 2019). By leveraging GEM of *K. pneumoniae* in host-like conditions in combination with *in silico* structure-based drug screening, these investigators identified several candidate molecules potentially targeting 2-dehydro-3-deoxyphosphooctonate aldolase activity; the gene coding for this enzyme, *kdsA*, is present in 100% of pathogenic *K. pneumoniae* strains and also considered an essential gene (Ramos et al., 2018). However, these predictions remain to be validated experimentally by using *in vitro* and/or *in vivo* models where the essentiality of the *kdsA* gene is tested by inactivating this locus (if possible) and also by verifying the impact of the identified molecules on the viability (or virulence traits) of *K. pneumoniae* species.

Similar approaches have also been used to study fungi. *Candida albicans* is a fungal opportunistic pathogen that is cause of increased mortality in patients suffering of candidiasis (Perlroth et al., 2007). As there are only a few classes of antifungal drugs that are used to treat *Candida* infections and an increase in strains presenting multi-drug resistant phenotypes have been observed (Arendrup and Patterson, 2017), new approaches must be developed to identify compounds that can be used as fungicidal agents *vs*. *Candida* species. Viana and colleagues reconstructed and validated a GEM of *C. albicans* (Viana et al., 2020) and they tested the *in silico* model for its capacity to predict essential genes in *C. albicans* that had been previously experimentally identified (O’Meara et al., 2015). The model was capable of accurately predicting 78% of the published essential genes and validated the already-known drug target gene ERG11 (Lupetti et al., 2002). Interestingly, the *C. albicans* protein Ura9 (a quinone-containing protein) was identified to be essential through modeling predictions; drugs such as Atovaquone, used to treat malarial infections caused by *Plasmodium falciparum*, could potentially be used to target Ura9. While not experimentally validated and the *in silico* predictions performed using Roswell Park Memorial Institute (RPMI) medium, which likely does not reflect the infection microenvironment in the host, the multiple targets identified through the GEM of *C. albicans* might represent novel targets for drug development or repurposing.

Metabolic modeling has also been used to find potential drug targets for parasites causing diseases in humans (Curran et al., 2020). The parasitic worm *Brugia malayi* is one of the causes of lymphatic filariasis (also known as elephantiasis) and is transmitted through blood-sucking mosquitoes; infection with this parasite can result in the swelling of limbs via chronic leakage of lymph into tissues (Chandy et al., 2011). Current effective anti-parasite drug treatment is limited as these therapies do not target all the life-stages of *B. malayi*, and to be effective, the drug regimen must be performed during the whole lifespan of the adult worm, which has previously been reported to last as long as 15 years (Molyneux et al., 2014). Recent studies have indicated that targeting *Wolbachia*, an endosymbiotic microorganism implicated in *B. malayi* fitness and its reproduction cycle, might represent an new therapeutic avenue (Taylor et al., 2013). In an attempt to identify potential drugs aimed at *Wolbachia* that would result in its elimination of *B. malayi*, Curran and colleagues reconstructed a GEM of the worm and identified 102 essential metabolic reactions predicted to be essential for the survival of the worm (Curran et al., 2020). By filtering out metabolic reactions for which no experimental expression data are available as well as the pathways with homology to human metabolic reactions, *in silico* modeling predicted that the enzymes 1-deoxy-D-xylulose 5-phosphate reductoisomerase, fructose biphosphatase and an adenylate kinase represent potential *Wolbachia* enzymes to be targeted. Interestingly, the drugs Fosmidomycin and tenofovir (both clinically approved) specifically target those enzymes and were experimentally confirmed to reduce the abundance of *Wolbachia* in *B. malayi*, negatively impacting the worm (Curran et al., 2020).

Taken together, the studies presented in this section support the notion that the integration of experimental expression data in combination with metabolic modeling and *in silico* drug screening might represent a powerful tool to identify drugs that might already be clinically available for other indications and repurposed for new diseases. Future research integrating *in vivo-*like conditions with metabolic modeling will certainly allow for the expansion of the complex metabolic interactions occurring between the host and microbe for the identification of novel antimicrobials with increased activity. Such efforts will surely be facilitated by the construction of integrated microbe-host GEMs to better understand such interactions in the context of human health and disease (Bordbar et al., 2010; Thiele et al., 2020).

## Concluding Remarks

The microbial complexity existing in various ecological niches, including in the context of human infections or microbiomes, necessitates the utilization of novel approaches to probe key metabolic features governing the interactions among microbes and/or the host. We argue that metabolic modeling represents such a tool as it has proven its usefulness in conditions where experimental methods alone were not sufficiently efficient, as for assessing the production of complex mixtures of microbial-derived metabolites (Simeonidis and Price, 2015). Starting with single species and more recently expanding to complex polymicrobial interactions, metabolic modeling has been employed to tackle increasingly complex questions by combining GEMs with nutritional information reflecting the infection environment to pinpoint metabolic pathways driving community structure and metabolite cross-feeding (Bordbar et al., 2010; Thiele et al., 2020).

Perhaps one of the most compelling ecological niches wherein metabolic modeling is currently employed and for which the most data is available is the human gut. Metabolic interactions are likely critical to maintain homeostasis between polymicrobial communities and the host in the gut (Shoaie and Nielsen, 2014; Kumar et al., 2019). Metabolic modeling has also proven to be useful in the context of microbial-based human diseases such as CDI and CF, where key metabolic features have been predicted to drive the abundance of pathogens (Henson et al., 2019; Phalak and Henson, 2019). Obtaining such detailed metabolic understanding of biochemical interactions driving microbial community structure, especially in the context of complex polymicrobial diseases or human microbial communities, could help efforts focused at the identification of new drug targets or probiotic interventions. Furthermore, the development of spatiotemporal metabolic models capable of integrating additional variables such as nutrient and oxygen diffusion/availability (Phalak et al., 2016; Chan et al., 2019; Altamirano et al., 2020) will further improve the computational predictions regarding how microbial community structure and function is impacted in complex polymicrobial diseases.

Given that multiple chronic diseases such as periodontitis, CF and diabetic foot ulcers are driven by the presence of polymicrobial communities that exhibit recalcitrance to antimicrobials (Rams et al., 2014; O’Toole, 2018; Orazi and O'Toole, 2019; Heravi et al., 2020), the field is poised to apply combined modeling and experimental approaches to better understand how to effectively treat such polymicrobial infections. To date however, little or no efforts have been directed towards modeling these complicated, often chronic polymicrobial infections and their predicted responses to antibiotic treatment.

One limitation of the approaches discussed here is the lack of a systematic evaluation of multiple GEMs existing for a same organism. That is, several GEMs with variations in model annotation, reaction stoichiometry, or the presence/absence of cofactors, can exist for a given organism but no unanimously standardized approach for the construction of a GEM has been adopted to date (Lieven et al., 2020). This lack of standardization can in turn lead to inconsistent predictions and a lack of reproducibility among models and laboratories. For instance, in an attempt to identify the best performing GEM for *Mycobacterium tuberculosis* among eight such recently published models, Lopez and colleagues observed that only two of the GEMs were able to generate robust predictions when using parameters such as the number of reaction gaps in the metabolic network, thermodynamically infeasible reactions, dead-end metabolites and gene essentiality predictions using previously published experimental data (Lopez-Agudelo et al., 2020). To remedy such potential shortcomings in the future utilization of GEMs, MEMOTE (MEtabolic MOdel TEsts) an open-source software driven by a community effort, has been published by Lieven and colleagues (Lieven et al., 2020). MEMOTE helps in improving GEM reproducibility and reuse among studies by advocating the utilization of a standardized GEM data format exchange, that is, the Systems Biology Markup Language level 3 flux balance constraints (SBML3FBC) which include defined parameters such as metabolite chemical formulas, charge and annotations, etc. (Lieven et al., 2020). Furthermore, by examining factors including biomass reaction, stoichiometry inconsistencies, annotation validation and basic tests, MEMOTE has the capacity to benchmark GEMs that are currently available throughout several databases, thus helping in the selection and continuous improvement of metabolic models.

Ultimately, we argue that metabolic modeling will prove quite valuable assisting experimentalists to focus their research efforts on a specific sets of questions originating from clinical observations by helping in the identification of key metabolic pathways likely responsible for driving disease. To validate the data from metabolic modeling studies examining complex microbial communities in the context of microbiomes or mixed-species infections, the development of more complex *in vitro/in vivo* communities composed of abundant and prevalent species will be necessary to better probe how microbe-microbe and polymicrobial community-host metabolic interactions are impacting community structure and function, including the capability of these communities to drive negative clinical outcomes. We also envision that the integration of various *in vivo*-like environmental factors such as oxygen and nutrient diffusion/availability, pH, viscosity, etc. will be critical components to be integrated in experimental models to help in our understanding of how metabolic interactions drive community structure and function. For instance, the development of a an artificial sputum medium developed by the Whiteley group, which was formulated based on nutrients available in the CF airway, (e.g. lactate, amino acids, dextrose, etc.) and mimicking the viscosity observed in the lung environment has allowed for significant breakthroughs in CF research (Palmer et al., 2005; Palmer et al., 2007; Turner et al., 2015; Darch et al., 2018; Cornforth et al., 2020). One could argue that the creation of disease-like growth media for the gut will also result in the discovery of novel findings impacting human health.

In summary, we believe that multi-omics data, (i.e. amplicon, metagenomic, metabolomic, transcriptomic) represent critical information regarding the infection environment that will drive the construction of robust laboratory models, which in combination with metabolic modeling, will trigger an iterative process between computational predictions and laboratory validation to improve our understanding of community structure and function and to facilitate the identification of novel therapeutics.
